# Solid variant of aneurysmal bone cyst of the heel: a case report

**DOI:** 10.1186/1752-1947-5-145

**Published:** 2011-04-12

**Authors:** Joanna A Lekka, Theofani V Gavresea, Gabriela A Stanc-Giannakopoulos, Nikolaos S Demertzis

**Affiliations:** 1 Department of Pathology, Metaxas Anticancer Hospital, Piraeus, Greece; 2 Department of Orthopaedic Surgery, Metaxas Anticancer Hospital, Piraeus, Greece

## Abstract

**Introduction:**

An aneurysmal bone cyst is a benign but often rapidly expanding osteolytic multi-cystic osseous lesion that occurs as a primary, secondary, intra-osseous, extra-osseous, solid or conventional lesion. It frequently coexists with other benign and malignant bone tumors. Although it is considered to be reactive in nature, there is evidence that some aneurysmal bone cysts are true neoplasms. The solid variant of aneurysmal bone cyst is a rare subtype of aneurysmal bone cyst with a preponderance of solid to cystic elements. Such a case affecting the heel, an unusual site, is reported.

**Case presentation:**

A 26-year-old Caucasian man presented with pain and swelling in his left lower extremity. A plain radiograph demonstrated an intra-osseous, solitary, eccentric mass in the front portion of the left heel. Computed tomography and magnetic resonance imaging scans showed that the lesion appeared to be sub-cortical, solid with a small cystic portion without the characteristic fluid-fluid level detection but with distinct internal septation. Bone images containing fluid-fluid levels are usually produced by aneurysmal bone cysts. The fluid-fluid level due to bleeding within the tumor followed by layering of the blood components based density differences, but it was not seen in our case. An intra-lesional excision was performed. Microscopic examination revealed fibrous septa with spindle cell fibroblastic proliferation, capillaries and extensive areas of mature osteoid and reactive woven bone formation rimmed by osteoblasts. The spindle cells had low mitotic activity, and atypical forms were absent. The histological features of the lesion were consistent with the solid variant of an aneurysmal bone cyst.

**Conclusion:**

Solid aneurysmal bone cysts have been of great interest to pathologists because they may be mistaken for malignant tumors, mainly in cases of giant cell tumors or osteosarcomas, because of cellularity and variable mitotic activity. It is rather obvious that the correlation of clinical, radiological and histological findings is necessary for the differential diagnosis. The eventual diagnosis is based on microscopic evidence and is made when a predominance of solid to cystic elements is found. The present case is of great interest because of the nature of the neoplasm and the extremely unusual location in which it developed. Pathologists must be alert for such a diagnosis.

## Introduction

An aneurysmal bone cyst (ABC) is a benign, often rapidly expanding, locally destructive cystic lesion of the bone. Although ABC affects all age groups, the peak incidence is in the first and second decades of life. It accounts for about 1% to 2% of biopsied primary bone tumors. It may be of conventional or solid type [1-3]. The solid variant is a rare sub-type, and it is more difficult to recognize. The term "giant cell reparative granuloma" has been used as a synonym in the pathology literature to describe this variant. The metaphyseal region of long bones, including the distal femur and the proximal tibia, are the most common sites. The cervical spine also is frequently involved. An ABC may arise *de novo *as a primary ABC or secondarily in association with other benign and malignant bone tumors. Giant cell tumors, osteoblastomas, chondroblastomas and fibrous dysplasias are the most common benign neoplasms [2,4]. Although the cause of ABC is unknown, a reactive process after a fracture or as the result of arteriovenous malformation has been suggested. More recent cytogenetic studies have addressed the neoplastic nature of some ABCs that is due to a specific chromosomal abnormality [2,4-6], although ABCs traditionally have been regarded as non-neoplastic.

## Case presentation

**Figure 1:**
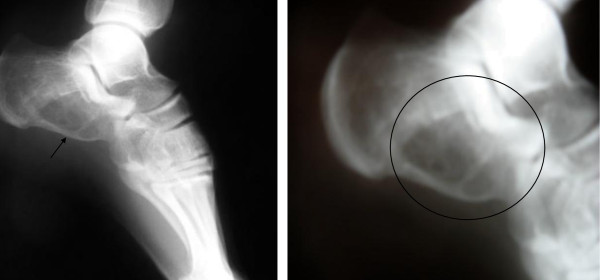
**Radiograph showing an expansive but well-marginated osteolytic lesion in the front portion of the patient's left heel**.

**Figure 2:**
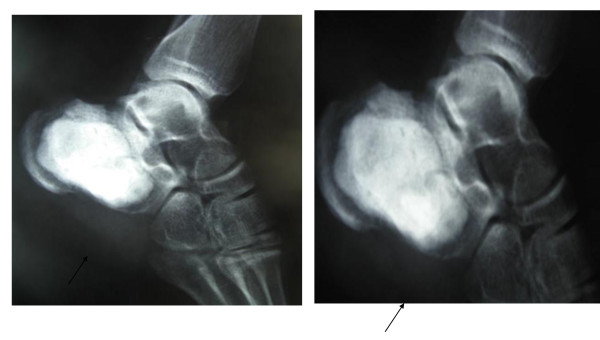
**Reconstruction of the gap with poly(methyl methacrylate) after surgical removal of the solid ABC**.

A 26-year-old Caucasian man presented with pain and swelling of his left lower extremity. A plain radiograph demonstrated an intra-osseous, solitary, eccentric mass in the front portion of the left heel. It was 2 cm in maximum diameter, destructive and expansile, but it was also a well-demarcated osteolytic lesion. On computed tomography and magnetic resonance imaging (MRI) scans, the lesion appeared to be sub-cortical, solid with a small cystic portion without the characteristic fluid-fluid levels detection, but with distinct internal septation (Figure 1). Bone images containing fluid-fluid levels are usually produced by aneurysmal bone cysts. The fluid-fluid level due to bleeding within the tumor followed by layering of the blood components based density differences, but it was not seen in our case. The cortex was of varying thickness and focally destroyed, without a peripheral outer shell of bone. An intra-lesional excision was performed. The gap was reconstructed with poly(methyl methacrylate) (Figure 2). There was no sign of local recurrence two years after surgery.

**Figure 3:**
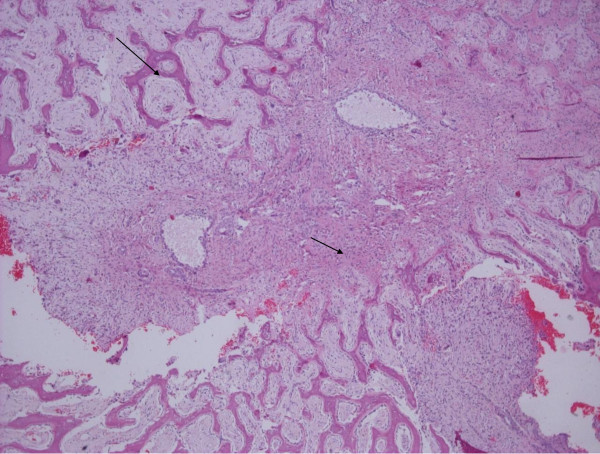
**Spindle cell proliferation (small arrow) and reactive woven bone formation (large arrow) rimmed by osteoblasts**. There were also small aneurysmal cystic spaces. Hematoxylin and eosin stain; original magnification, ×40.

**Figure 4:**
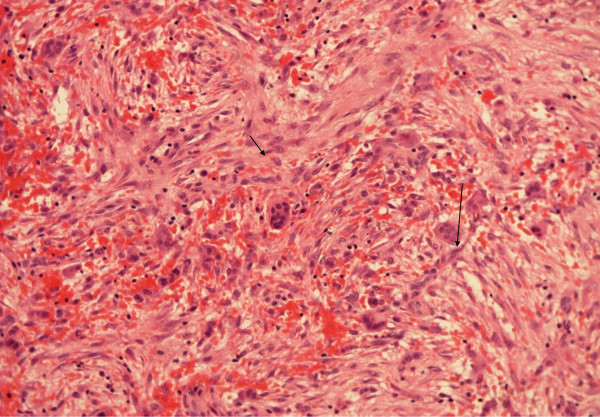
**Fibrous septa with spindle cells (small arrow) and multinucleated giant cells (large arrow)**. No evidence of malignancy is seen. Hematoxylin and eosin stain; original magnification, ×100.

**Figure 5:**
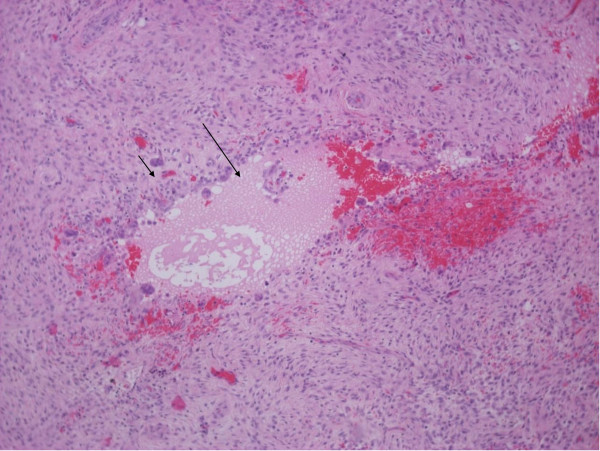
**Small aneurysmal cystic space (large arrow) and fibrous septa with spindle cells and multinucleated giant cells (small arrow)**. Hematoxylin and eosin stain; original magnification, ×40.

The curetted lesion was resected in sufficiently solid, granular, reddish-brown fragments. Microscopic examination revealed fibrous septa with spindle cell fibroblastic proliferation, capillaries and extensive areas of mature osteoid and reactive woven bone formation rimmed by plump benign-appearing osteoblasts (Figures 3 and 4). The bony trabeculae tended to anastomose. The spindle cells had low mitotic activity, and atypical forms were absent. There were no signs of nuclear atypia, necrosis or other features of malignancy. Multi-nucleated giant cells were patchy in distribution. There were also sparse, small, aneurysmal cystic spaces identified that contained blood and were surrounded by multi-nucleated giant cells (Figure 5). Scattered histocyte-like cells and lymphocytic foci were observed. The histological features were consistent with the solid variant of an ABC.

## Discussion

Solid ABC is a rare ABC variant with the same or similar clinical and radiologic features as a conventional lesion of an ABC, but with the absence of microscopic evidence of a cystic component or a preponderance of solid to cystic elements. In 1942, Jaffe and Lichtenstein [7] were the first to describe ABC. In 1983, Sanerkin *et al. *[8] described a variant of ABC in which the predominant histological features were solid, and they used the term "solid variant of aneurysmal bone cyst" to describe it. Jaffe in 1953 [9] and Lorenzo and Dorfman in 1980 [10] used the term "giant cell reparative granuloma" to describe non-neoplastic hemorrhagic processes in the jaw and the short tubular bones of the feet and hands. Ackerman and Spjut in 1962 [11] also described two lesions in the phalanges as a "giant cell reaction."

The histological findings of solid ABC and giant cell reparative granuloma are very similar, if not identical, and these terms are used synonymously in the literature, although some authors have criticized the use of the term "solid cyst" as being confusing [5]. The most common sites of ABC are the long bones of the lower extremities and, less frequently, the spine. Solid ABC has been described in the posterior component of the vertebral bodies [12], in the mandible [13,14] and in unusual locations such as the right fourth rib [15], while giant cell reparative granuloma has also been described in the craniofacial bones [16] and the nasal cavity [17].

Solid ABCs have been of great interest to pathologists because they may be mistaken for malignant tumors, mainly in cases of giant cell tumors or osteosarcomas, on the basis of cellularity and variable mitotic activity. A giant cell tumor has a uniform distribution of the giant cells and diffuse arrangement of round to oval stromal cells. In ABCs, the giant cells are usually scattered or gathered in small clusters and only in rare instances may be abundant. The stromal cells are spindle-shaped without histologic evidence of sarcomatous transformation.

Low-grade osteosarcoma is much less cellular and less active mitotically, while high-grade osteosarcoma obviously shows marked pleomorphism. The pattern of reactive bone production is different from the type of bone that one expects to see in low-grade and high-grade osteosarcomas [1,4]. In ABCs, the reactive bonny trabeculae are lined by plump, benign-appearing osteoblasts, which are not a feature of osteosarcomas.

The most acceptable pathogenetic hypothesis for ABC is some local circulatory disturbance leading to markedly increased venous pressure and the development of a dilated and enlarged vascular mass within the affected bone area. Although ABCs are known to be non-neoplastic and probably reactive lesions, more recent studies have shown that at least some of the ABCs of all types are of a neoplastic nature, owing to clonal chromosomal aberrations [6,18,19]. Recent cytogenetic data have shown clonal rearrangements of chromosomal bands 16q22 and 17p13, indicating a neoplastic basis in at least some ABCs [5,6,19].

## Conclusion

Solid ABCs have been of great interest to pathologists because they may be mistaken for malignant tumors, mainly giant cell tumors or osteosarcomas, because of cellularity and variable mitotic activity. It is rather obvious that the correlation of clinical, radiological and histological findings is necessary for the differential diagnosis.

In clinical practice, it is important to recognize that these lesions are benign conditions, and surgical excision, usually with curettage, is the treatment of choice. The differential diagnosis of ABCs from osteosarcomas is the most important clinical aspect because osteosarcomas must be treated with surgical excision and chemotherapy and have a different prognosis and incidence of recurrence.

Radiologists, surgeons and pathologists need to be aware of the wide range of imaging features, the unusual locations and the microscopic difficulties that may be encountered in cases of ABCs.

When MRI is performed and solid cystic elements are identified in an osteolytic lesion, the diagnosis of a solid ABC may be considered. It is rather obvious that the correlation of clinical, radiological and histological findings is necessary for the differential diagnosis. The eventual diagnosis is based on microscopic evidence and is made when a predominance of solid to cystic elements is found.

## Competing interests

The authors declare that they have no competing interests.

## Consent

Written informed consent was obtained from the patient for publication of this case report and any accompanying images. A copy of the written consent is available for review by the Editor-in-Chief of this journal.

## Authors' contributions

JL performed the histological examination and wrote the manuscript. TVG was a major contributor in writing the manuscript and helped with the figures. GG helped with the immunohistochemical study. ND performed the surgery and the gap reconstruction. All authors read and approved the final manuscript.
